# Synthesis, ADMET Properties, and In Vitro Antimicrobial and Antibiofilm Activity of 5-Nitro-2-thiophenecarbaldehyde N-((E)-(5-Nitrothienyl)methylidene)hydrazone (KTU-286) against *Staphylococcus aureus* with Defined Resistance Mechanisms

**DOI:** 10.3390/antibiotics9090612

**Published:** 2020-09-17

**Authors:** Povilas Kavaliauskas, Birute Grybaite, Vytautas Mickevicius, Ruta Petraitiene, Ramune Grigaleviciute, Rita Planciuniene, Philip Gialanella, Alius Pockevicius, Vidmantas Petraitis

**Affiliations:** 1 Division of Infectious Diseases, Department of Medicine, Weill Cornell Medicine of Cornell University, New York, NY 10065, USA; rop2016@med.cornell.edu (R.P.); vip2007@med.cornell.edu (V.P.); 2 Institute of Infectious Diseases and Pathogenic Microbiology, 59115 Prienai, Lithuania; 3 Biological Research Center, Lithuanian University of Health Sciences, 47181 Kaunas, Lithuania; ramune.grigaleviciute@lsmuni.lt; 4 Department of Organic Chemistry, Kaunas University of Technology, 50254 Kaunas, Lithuania; birute.grybaite@ktu.edu (B.G.); vytautas.mickevicius@ktu.lt (V.M.); 5 Institute of Microbiology and Virology, Lithuanian University of Health Sciences, 47181 Kaunas, Lithuania; rita.planciuniene@lsmuni.lt; 6 Department of Pathology, Montefiore Medical Center and Albert Einstein College of Medicine, New York, NY 10467, USA; pgialane@montefiore.org; 7 Department of Veterinary Pathobiology, Lithuanian University of Health Sciences, 47181 Kaunas, Lithuania; alius.pockevicius@lsmuni.lt

**Keywords:** bisnitrothiophene, *Staphylococcus aureus*, MRSA, VRSA, antimicrobial, small molecules, thiophene derivatives, drug resistant, hydrazone

## Abstract

The emergence of drug-resistant *Staphylococcus aureus* is responsible for high morbidity and mortality worldwide. New therapeutic options are needed to fight the increasing antimicrobial resistance among *S. aureus* in the clinical setting. We, therefore, characterized the in silico absorption, distribution, metabolism, elimination, and toxicity (ADMET) and in vitro antimicrobial activity of 5-nitro-2-thiophenecarbaldehyde N-((E)-(5-nitrothienyl)methylidene)hydrazone (KTU-286) against drug-resistant *S. aureus* strains with genetically defined resistance mechanisms. The antimicrobial activity of KTU-286 was determined by CLSI recommendations. The ADMET properties were estimated by using in silico modeling. The activity on biofilm integrity was examined by crystal violet assay. KTU-286 demonstrated low estimated toxicity and low skin permeability. The highest antimicrobial activity was observed among pan-susceptible (Pan-S) *S. aureus* (minimal inhibitory concentration (MIC) 0.5–2.0 µg/mL, IC_50_ = 0.460 µg/mL), followed by vancomycin resistant *S. aureus* (VRSA) (MIC 4.0 µg/mL, IC_50_ = 1.697 µg/mL) and methicillin-resistant *S. aureus* (MRSA) (MIC 1.0–16.0 µg/mL, IC_50_ = 2.282 µg/mL). KTU-286 resulted in significant (*p* < 0.05) loss of *S. aureus* biofilm integrity in vitro. Further studies are needed for a better understanding of safety, synergistic relationship, and therapeutic potency of KTU-286.

## 1. Introduction

*Staphylococcus aureus* is one of the most common causes of severe nosocomial infections worldwide [1]. Infections caused by drug-resistant *S. aureus* pose a huge threat to public health. The emergence of drug resistance among *S. aureus* decreases the number of potential therapeutic options, resulting in higher morbidity and mortality. New antimicrobial agents are needed to overcome rapidly rising drug resistance among *S. aureus* [2].

Methicillin-resistant *S. aureus* (MRSA) is one of the major causes of community- and hospital-acquired infections that are not restricted geographically and are distributed worldwide [3]. The major molecular determinants resulting in the MRSA phenotype and resistance to *β*-lactam antibiotics are located in *S. aureus* chromosome cassettes carrying *mecA* or *mecC* genes. Transpeptidase PB2a, encoded by *S. aureus mecA* or *mecC*, lowers the affinity of the penicillin-binding protein (PBP) to *β*-lactam antibiotics. The loss of PBP affinity leads to *S. aureus* resistance to the acquisition of multidrug resistance in *S. aureus* [4].

Various glycopeptides are often used for the treatment of infections caused by different Gram-positive organisms. Complicated and life-threatening infections caused by drug-resistant *S. aureus* are often treated with glycopeptide drug vancomycin as a primary treatment option [5]. The resistance of *S. aureus* to vancomycin is mediated via acquisition of the gene cluster encoding *vancA* from *Enterococcus* spp. The resistance genes are located in *Tn*1546 transposon and transferred via horizontal gene transfer. Many strains of vancomycin resistant *S. aureus* (VRSA) often share resistance to large variety of other antimicrobials due to co-acquisition of multiple resistance genes, such as *ermA, imrB,* and *mecl* [6]. The increasing global spread of highly resistant and invasive VRSA strains requires development of new treatment options.

*S. aureus* express the variety of adhesins and virulence-associated factors that are important for adhesion, biofilm formation, and host colonization. Various *S. aureus*-encoded virulence factors (SasG, protein A, Clf, SdrC, FnBPA) are important for *S. aureus* biofilm formation and host colonization [7,8]. Numerous studies have showed that *S. aureus* biofilm formation increases upon exposure to various antimicrobials. Therefore, inadequate or incomplete antimicrobial therapy could promote *S. aureus* biofilm formation, consequentially worsening the infection prognosis [9]. Finally, many *S. aureus* biofilm associated infections are often of polymicrobial nature, which increases the protection of biofilm-associated pathogens from antimicrobials [10,11].

Novel therapeutic approaches that target *S. aureus* quorum sensing (QS) signaling, a key regulator of virulence and biofilm formation, are being widely explored. For instance, *trans*-farnesol was explored by several studies as biofilm formation modulator [11,12]. Although *trans*-farnesol was showed to be active *S. aureus* biofilm disrupting agent, the further studies of Kong et al. report that *trans*-farnesol act as a strong *S. aureus* vancomycin efflux pump inducer, further promoting resistance among *S. aureus* [11].

Heterocyclic compounds are extensively distributed in nature and have versatile synthetic applicability and biological activity [13]. Thiophene derivatives are a considerable group of compounds with a promising therapeutic effect against various diseases [14]. A number of studies have shown that thiophene-ring-containing derivatives display antifungal, antiviral, antibacterial, anti-inflammatory, biofilm-disrupting and cytostatic activity [15,16,17,18,19]. The wide spectrum of biological activity of thiophenes makes them attractive building blocks for synthesis and development of novel biologically active compounds that have promising antimicrobial and antibiofilm activity.

Herein, we report the synthesis, in silico pharmacological characterization, and assessment of in vitro antimicrobial activity of 5-nitro-2-thiophenecarbaldehyde N-((E)-(5-nitrothienyl)methylidene)hydrazone (KTU-286) with selective activity against *Staphylococcus aureus* with defined, emerging resistance mechanisms.

## 2. Results

### 2.1. Synthesis and Characterization of 5-Nitro-2-thiophenecarbaldehyde N-((E)-(5-Nitrothienyl)methylidene)hydrazone (KTU-286)

**Figure 1:**
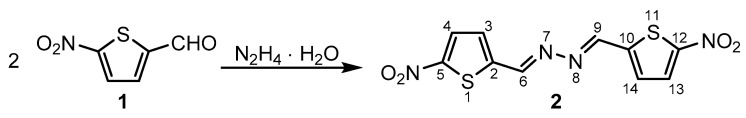
Synthesis of 5-nitro-2-thiophenecarbaldehyde *N*-((*E*)-(5-nitrothienyl)methylidene)hydrazone (KTU-286).

The 5-nitro-2-thiophenecarbaldehyde *N*-[(*E*)-(5-nitrothienyl)methylidene)hydrazone **(2)** (KTU-286) was synthesized according the Figure 1.

A mixture of 5-nitrothiophene-2-carboxaldehyde **1** (0.55 g, 3.5 mmol), hydrazine hydrate (0.09 g, 1.8 mol), and 2-propanol (15 mL) was stirred at reflux for 1 h. Crystalline product **2** (KTU-286) was filtered off, washed with 2-propanol, and dried.

Yellow powder, yield 0.95 g (87%), m.p. 214–216 °C; ^1^H-NMR (400 MHz, DMSO-d_6_) δ: 7.07 (d, J = 4.3 Hz, 2H, CH), 7.80 (s, 2H, CH=N), 7.99 (d, J = 4.3 Hz, 2H, CH); ^13^C-NMR (400 MHz, DMSO-d_6_) δ: 123.4 (3, 14), 129.4 (4, 13), 131.2 (6, 9), 147.0 (2, 10), 151.5 (5, 12); Anal Calcd for C_10_H_6_N_4_O_4_S_2_, %: C, 38.71, H, 1.95, N, 18.06. Found: C, 38.83, H, 2.01, N, 18.01.

### 2.2. KTU-286 Demonstrated Low Predicted CNS and Skin Permeability and Good GI Absorption Characteristics

In silico structure-based pharmacological prediction was used to describe major pharmacological characteristics of the 5-nitro-2-thiophenecarbaldehyde *N*-((*E*)-(5-nitrothienyl)methylidene)hydrazone (KTU-286). The computational analysis showed relatively low solubility in water (−4.724 log mol/L), moderate Caco-2 intestinal permeability (0.932 cm/s), and high gastrointestinal (GI) absorption (94.139% of GI absorption). The predicted GI absorption of KTU-286 was greater than vancomycin. In silico prediction demonstrated that KTU-286 is poorly permeable through blood–brain barrier (BBB) (−1.09 log BB) and has low permeability in central nervous system (−2.249 log PS) and skin (−2.642 log Kp) (Table 1).

The compound was not found to be a substrate or inhibitor of P-glycoprotein I and II, suggesting that the transportation and excretion of the 5-nitro-2-thiophenecarbaldehyde *N*-((*E*)-(5-nitrothienyl)methylidene)hydrazone (KTU-286) is not dependent on the P-glycoprotein pathway.

Collectively, our in silico prediction results demonstrate that KTU-286 exhibits limited permeability characteristics, suggesting a possible application niche as a topical antimicrobial agent.

### 2.3. KTU-286 Metabolism Is Dependent on CYP3A4 Cytochrome and Is Predicted to Be Excreted via Kidneys

After evaluating characteristics of compound permeability through various barriers we further predicted the KTU-286 and vancomycin (used as control) excretion kinetics and molecular interactions with human cytochrome systems.

In silico analysis revealed that both KTU-286 and vancomycin were not substrates of human OCT2, which is important for the excretion of cationic molecules. In silico estimation predicted that KTU-286 is excreted moderately via kidneys (0.479 log mL/min/kg), with low steady-state volume distribution (VD_ss_) (Table 2).

To better understand the metabolism pathways of KTU-286, we further examined in silico interactions of KTU-286 and human cytochrome systems known to be responsible for drug metabolism and excretion and compared it with vancomycin. KTU-286 was found to be a substrate of CYP3A4, but not CYP2D6 cytochromes. KTU-286 was predicted to be an inhibitor of CYP1A2, CYP2C19, and CYP2C9 cytochromes. CYP2D6 and CYP3A4 were not inhibited by the compound in silico (Table 3). No interactions with major human cytochromes were predicted when vancomycin was used for the in silico predictions.

### 2.4. KTU-286 Shows Low Predicted Cytotoxicity, Hepatotoxicity, and Immunotoxicity

The pkCSM and ProTox-II software were used for evaluation of toxicological parameters in silico. The predicted rat acute oral toxicity (LD_50_) of KTU-286 was 2.748 mol/kg, while chronic oral rat toxicity was 1.149 log mg/kg/day (Table 4). The predicted acute rat toxicity of KTU-286 was similar to that of vancomycin (2.482 mol/kg), while chronic oral rat toxicity of KTU-286 was greater than that of vancomycin (9.152 log/mg/Kg/day). The KTU-286 showed low predicted acute murine toxicity (LD_50_ = 750 mg/kg) (Table 4). The KTU-286 was not estimated to be hepatotoxic or immunotoxic, although an in silico AMES test indicated possible carcinogenicity of KTU-286.

To better understand the possible ecological impact of KTU-286, we applied in silico environmental toxicology models to predict the toxicity to *Tetrahymena pyriformis* and minnow larvae. In silico analysis of the KTU-286 toxicity against *T. pyriformis* and minnow larvae revealed the moderate environmental hazard of KTU-286. The estimated toxic dose for *T. pyriformis* was 2.125 µg/L, while the minnow toxic dose was estimated as 0.222 log mM (Table 4).

Collectively, our results show that KTU-286 exhibits higher predicted in silico toxicity in comparison to vancomycin, therefore limiting possible systemic applications of KTU-286 in the treatment of infections caused by *S. aureus.*

### 2.5. KTU-286 Is Predicted to Interact with Mitochondrial Membrane Potential Transduction Pathway

After establishing major toxicological properties in silico, we attempted to identify the KTU-286 toxicity-associated targets and predict the toxicity mechanisms of KTU-286. ProTox-II was used for molecular docking studies of KTU-286 with 12 major human toxicity-associated targets (Table 5).

The molecular docking studies performed using ProTox-II showed that KTU-286 interacts with the mitochondria-directed stress response pathway. Further analysis revealed that among potential stress response pathway targets, a mitochondrial membrane was identified as a significant KTU-286 toxicity target (Table 5).

### 2.6. Predicted Immunomodulatory Activity of KTU-286 Might Be Associated with Leukotriene Signaling Pathway

To further understand the pharmacological activity of KTU-286, we decided to estimate the probable human binding targets of KTU-286. The in silico analysis was performed using SwissTargetPrediction software to screen the library of human protein targets against the structure of KTU-286.

**Figure 2:**
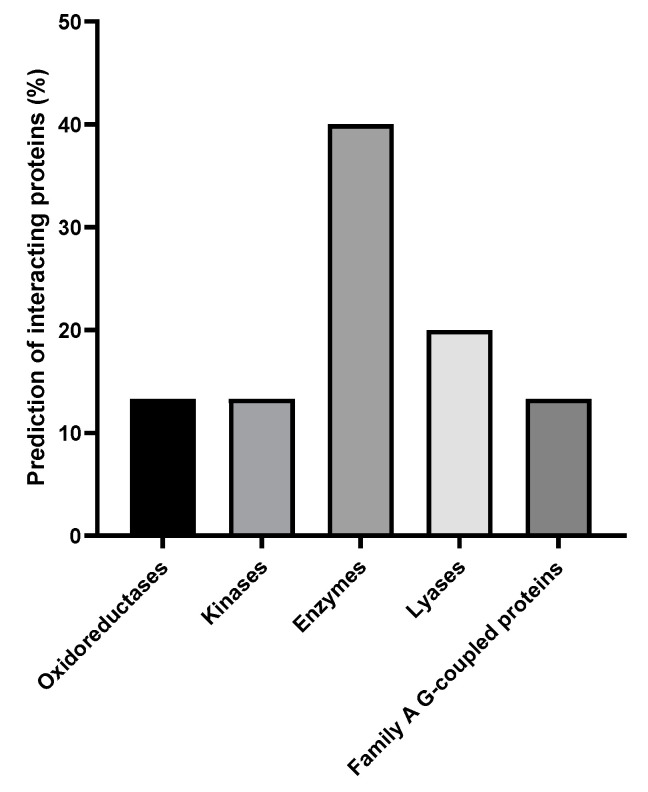
KTU-286 is predicted to interact with five different human protein classes. The in silico interactions were computed by using SwissTargetPrediction software, and the generated percentages of human proteins interacting with KTU-286 were used. Among identified interacting targets, 40% were proteins with unspecified enzymatic properties, 20% were various lyases, 13.3% were various kinases, 13.3% were various oxidoreductases, and 13.3% were various family A G-coupled proteins.

KTU-286 exhibited the predicted interactions with 31 human target proteins with interaction probability of 0.1005 (Table S2). Among identified interacting targets, 40% were proteins with unspecified enzymatic properties; 20% were various lyases, 13.3% were various kinases, 13.3% were 13.3% were various oxidoreductases, and 13.3% were family A G-coupled proteins (Figure 2).

KTU-286 showed predicted interactions with human arachidonate 5-lipoxygenase (ALOX5), prostaglandin E synthase (PTGES), and interleukin-8 receptor B (CXCR2). All major identified members (*n* = 3) are involved in the leukotriene signaling pathway, suggesting possible immunomodulatory or anti-inflammatory properties of KTU-286. Further experimental studies are needed to validate the predicted targets.

### 2.7. Selective Antibacterial Activity of KTU-286 against S. aureus

After characterizing absorption, distribution, metabolism, elimination, and toxicity (ADMET) properties of 5-nitro-2-thiophenecarbaldehyde *N*-((*E*)-(5-nitrothienyl)methylidene)hydrazone (KTU-286), we further determined the antimicrobial activity of KTU-286 against a collection of clinically relevant fungal and bacterial pathogens using in vitro experimental approaches. KTU-286 demonstrated good and selective antimicrobial activity against *S. aureus.* Moreover, KTU-286 showed slight anti-mycobacterial activity against *Mycobacterium abscessus* (minimal inhibitory concentration (MIC) 64 µg/mL)*,* but not against *Mycobacterium smegmatis* (MIC < 128 µg/mL) or *Mycobacterium bovis* BCG (MIC < 128 µg/mL). No antifungal activity was observed when KTU-286 was tested against fungal pathogens (Table S1).

To further characterize the antibacterial activity against *S. aureus* and determine whether existing emerging resistance mechanisms play a role in *S. aureus* susceptibility to KTU-286, we used a panel of *S. aureus* with genetically defined resistance mechanisms. Furthermore, we compared KTU-286 MIC values with antimicrobial activity (MIC) of vancomycin, a common antibiotic used for treatment of infections caused by *S. aureus* (Table 6).

5-Nitro-2-thiophenecarbaldehyde *N*-((*E*)-(5-nitrothienyl)methylidene)hydrazone showed good antibacterial activity against pan-susceptible *S. aureus,* with MIC_50_ of 0.5 µg/mL and MIC_90_ of 1 µg/mL (Table 6). Among all tested pan-susceptible (Pan-S) *S. aureus* strains (*n* = 40), 45% (*n* = 18) had MIC of 1 µg/mL, and 37.5% (*n* = 15) of strains had MIC of 0.5 µg/mL. Finally, 17.5% of Pan-S *S. aureus* had MIC of 2 µg/mL. The MIC for vancomycin among tested Pan-S *S. aureus* strains was 0.5–1.0 µg/mL (Table 6).

MRSA strains carrying SC*mecA* showed high variation in MIC distribution of KTU-286. The measured MIC_50_ was 1–2 µg/mL, while MIC_90_ of 4 µg/mL was observed among all tested MRSA isolates. Among all analyzed MRSA isolates, 20% (*n* = 6) showed MIC of 1 µg/mL, 6.7% (*n* = 2) had MIC of 2 µg/mL, and 40% (*n* = 12) demonstrated MIC of 4 µg/mL. Among the analyzed MRSA isolates, 13.3% (*n* = 4) had MIC of 8 µg/mL, and 20% (*n* = 6) showed MIC values of 16 µg/mL, demonstrating presumably MRSA-specific tolerance mechanisms. We further evaluated MRSA MIC values for vancomycin, a standard care option to treat *S. aureus* infections caused by MRSA. Among analyzed MRSA isolates, we found low *S. aureus* resistance to vancomycin. Vancomycin MIC values among tested isolates were 1–2 µg/mL (Table 6).

KTU-286 showed fixed MIC_50_, MIC_90_, and MIC values of 4 µg/mL observed among all (*n* = 30) analyzed *vancA*-carrying VRSA isolates, while MIC for vancomycin ranged from 8 to 34 µg/mL (Table 6).

These results demonstrate that KTU-286 exhibits selective activity against *S. aureus*, showing the highest antibacterial activity against Pan-S and VRSA *S. aureus* strains. Most importantly, the MIC of KTU-286 against VRSA was three to four times lower than the MIC of vancomycin, demonstrating good antibacterial activity against highly multidrug-resistant *S. aureus.*

### 2.8. Dose-Dependent Antimicrobial Activity of KTU-286 against S. aureus

After establishing the distribution of KTU-286 MIC among libraries of *S. aureus* with genetically defined resistance mechanisms, we further evaluated the in vitro kinetics of antimicrobial activity by using well-characterized, representative *S. aureus* isolates (Table 7). The isolates were selected to represent the major emerging resistance mechanisms observed among *S. aureus*. We used a modified spectrophotometric assay to evaluate the influence of 5-nitro-2-thiophenecarbaldehyde *N*-((*E*)-(5-nitrothienyl)methylidene)hydrazone on *S. aureus* growth inhibition.

**Figure 3:**
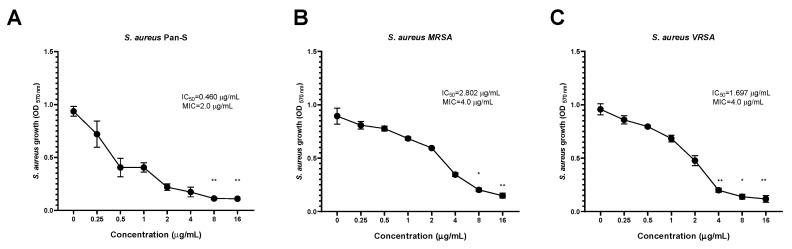
Concentration-dependent antibacterial activity of KTU-286 against *Staphylococcus aureus* with genetically defined resistance. Representative strains of pan-susceptible *S. aureus* (*blaZ*) (**A**), methicillin-resistant *S. aureus* (MRSA) (SC*mecA, ermA*) (**B**), and VRSA (*vanA, aac(6′)-aph(2″), tetK*) (**C**) were exposed to increasing concentrations of KTU-286 (0–16.0 µg/mL). The highest inhibitory activity of KTU-286 against *S. aureus* was also observed among pan-susceptible (Pan-S) *S. aureus* (IC_50_ = 0.460 µg/mL), followed by VRSA (IC_50_ = 1.697 µg/mL) and MRSA (IC_50_ = 2.802 µg/mL). Data represent mean ± SD of triplicate experiments (*n* = 3). Statistical differences were determined using Kruskal–Wallis test. * *p* < 0.05; ** *p* < 0.01.

The highest inhibitory activity of KTU-286 against *S. aureus* was also observed among Pan-S *S. aureus* (IC_50_ = 0.460 µg/mL), followed by VRSA (IC_50_ = 1.697 µg/mL) and MRSA (IC_50_ = 2.802 µg/mL), suggesting the possible existence of a yet unknown MRSA-specific KTU-286 tolerance mechanism (Figure 3, Table 7).

KTU-286 was able to significantly (*p* < 0.05) decrease the growth of *S. aureus* after 18 h of incubation. The highest growth reduction of *S. aureus* was observed among Pan-S at the concentrations of 8.0 and 16.0 µg/mL (*p* < 0.01), followed by VRSA and MRSA (Figure 3, Table 7).

We then compared antibacterial activity (MIC) of KTU-286 with antibacterial activity of vancomycin (MIC) against the same representative strains of *S. aureus*. The MIC of vancomycin observed against Pan-S *S. aureus* was 0.5 µg/mL, while MIC values for MRSA and VRSA were 1.0 and 32.0 µg/mL respectively. Interestingly, KTU-286 showed four times lower MIC against VRSA (MIC 4.0 µg/mL) in comparison to vancomycin (MIC 32.0 µg/mL), suggesting the possible existence of selective targets among VRSA.

Collectively, these results demonstrate the concentration-dependent activity of KTU-286 against *Staphylococcus aureus* harboring genetically defined resistance mechanisms and suggests the potential application of KTU-286 as a VRSA-targeting antimicrobial agent.

### 2.9. KTU-286 Is Bactericidal against S. aureus and Is Not Dependent on Existing Resistance

**Figure 4:**
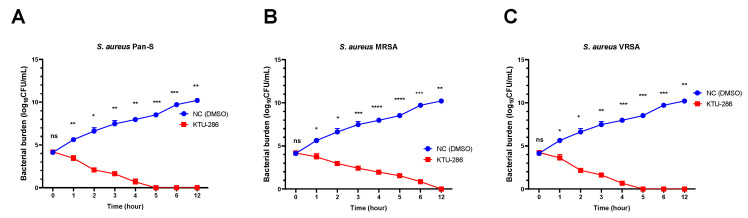
Time-kill kinetics of *Staphylococcus aureus*, showing bactericidal activity of KTU-286 against strains with genetically defined resistance. The Pan-S *Staphylococcus aureus* (**A**), MRSA (**B**), and VRSA (**C**) were exposed to MICs of KTU-286 (4.0, 8.0, and 4.0 µg/mL respectively). A significant reduction (*p* < 0.05) in *S. aureus* bacterial burden was observed among all *S. aureus* isolates used, without dependence on resistance mechanisms, when bacteria were exposed to the test compound for 12 h. Data represent mean ± SD of triplicate experiments (*n =* 3). Statistical differences were determined using two-way ANOVA test. * *p* < 0.05; ** *p* < 0.01; *** *p* < 0.001; **** *p* < 0.0001.

The time-kill assay was further used to determine the duration required to kill *S. aureus* and the type of KTU-286-mediated killing of *S. aureus.* For the time-kill assay, we prepared fixed MICs of KTU-286 and exposed representative *S. aureus* Pan-S (2.0 µg/mL), MRSA (8.0 µg/mL) and VRSA (4.0 µg/mL) to the test compound for 12 h (Figure 4).

A significant reduction (*p* < 0.05) in *S. aureus* bacterial burden was observed among all *S. aureus* isolates tested, without dependence on the resistance mechanisms, when bacteria were exposed to the test compound for 12 h. On average, the maximum reduction of bacterial burden was observed over the period of 4–6 h. *S. aureus* were not recovered from KTU-286 treated groups after 12 h of incubation, demonstrating strong bactericidal activity (Figure 4).

### 2.10. KTU-286 Significantly Impairs S. aureus Biofilm Integrity

It was previously demonstrated that several thiophene derivatives are capable of affecting bacterial biofilm integrity by possibly acting on bacterial quorum sensing. Therefore, we investigated whether 5-nitro-2-thiophenecarbaldehyde *N*-((*E*)-(5-nitrothienyl)methylidene)hydrazone is capable of affecting *S. aureus* biofilm integrity and if the biofilm-disrupting activity is dependent on the genetically defined resistance phenotype harbored by the isolates.

The *S. aureus* biofilms were grown for 48 h, and then matured biofilms were exposed with increasing concentrations (0.5 MIC, MIC, 2× MIC and 4× MIC) of KTU-286 for an additional 24 h. After exposure, the differences in *S. aureus* biofilm integrity were quantified.

**Figure 5:**
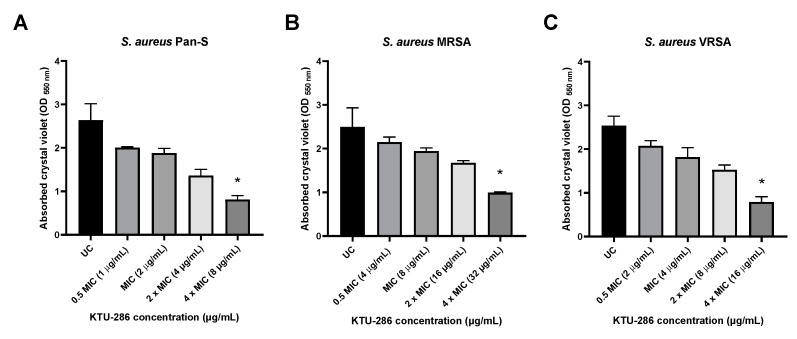
KTU-286 shows concentration-dependent *S. aureus* biofilm disrupting activity. Mature Pan-S (**A**), MRSA (**B**), and VRSA (**C**) biofilms were exposed to different concentrations of KTU-286 (0.5 MIC, MIC, 2× MIC and 4× MIC). Biofilm exposure to KTU-286 resulted in significant decrease of biofilm integrity (*p* < 0.05) in comparison to that of untreated control. Biofilm exposure to KTU-286 for 24 h resulted in a significant (*p* < 0.05) reduction in *S. aureus* biofilm integrity at the concentration four times higher than MIC. Data represent mean ± SD of triplicate experiments (*n =* 3). Statistical differences were determined using Kruskal–Wallis test. * *p* < 0.05.

Biofilm exposure to 5-nitro-2-thiophenecarbaldehyde *N*-((*E*)-(5-nitrothienyl)methylidene)hydrazone (KTU-286) resulted in a significant decrease of biofilm integrity (*p* < 0.05) in comparison to that of untreated control. Biofilm exposure to KTU-286 for 24 h resulted in a significant (*p* < 0.05) reduction in *S. aureus* biofilm integrity at the concentration four times higher than MIC. There were no significant differences observed between efficacy of biofilm reduction and the genetic resistance background of *S. aureus* (Figure 5).

Our results demonstrate that KTU-286 is capable of affecting *S. aureus* biofilm integrity at concentrations ten times higher than MIC. Further studies are needed to better understand the mechanism of *S. aureus* biofilm disruption by KTU-286 and the possible biofilm-targeting interactions of KTU-286 and other clinically available antimicrobials.

## 3. Discussion

The rising numbers of infections caused by multidrug-resistant *Staphylococcus aureus* are an important public health concern. Therefore, there is an urgent need to develop novel compounds with significant antimicrobial activity against highly resistant *S. aureus*. We synthesized and characterized the in vitro activity and in silico ADMET properties of 5-nitro-2-thiophenecarbaldehyde *N*-((*E*)-(5-nitrothienyl)methylidene)hydrazone (KTU-286). The synthesized compound is a nitrothiophene derivative with significant and selective bactericidal activity against multidrug-resistant *S. aureus* and its biofilms.

Various thiophene derivatives have been long known for their broad-spectrum biological activity, making these compounds useful candidates for the treatment of various diseases [Mabkhot, 2017 #38]. Despite their wide biological activity, the usage of compounds bearing thiophene moieties is often limited. Thiophene and its derivatives often show toxicity to animals and humans, and it is believed that this toxicity is associated with the production of highly active intermediates.

When administered in vivo, many thiophene-ring-containing compounds are oxidized by cytochrome P450, and the resulting thiophene-S-oxides, hydroxythiophene and mercapturic acid further modulate thiophene-associated toxicity [20] Moreover, some thiophenes have been demonstrated to be carcinogenic and genotoxic, further limiting their pharmacological application. Our results generated during in silico ADMET prediction showed limited and considerably low toxicity of KTU-286, making it a useful therapeutic candidate.

Synthesis and anti-plasmodium activity of 5-nitro-2-thiophenecarbaldehyde *N*-((*E*)-(5-nitrothienyl)-methylidene)hydrazone (KTU-286) were first described in a patent [21]. This compound was synthesized from 2-thiophene-5-nitrocarbaldehyde and hydrazine hydrate in tetrahydrofuran. The reaction solution was refluxed for two hours to give a 32% yield. The synthesis of the same compound, with antimycobacterial activity, was later described by Rando et al. [22]. The compound was obtained as a by-product from the synthesis of hydrazide derived from the nitrothienyl group and isoniazid. In our study, 5-nitro-2-thiophenecarbaldehyde *N*-((*E*)-(3-nitrothienyl)methylidene)hydrazone was synthesized in good 87% yield from 2-thiophene-5-nitrocarbaldehyde and hydrazine hydrate in 2-propanol. The reaction was carried out at reflux (Figure 1).

Various nitrothiophene derivatives sharing structural similarity to KTU-286 have been known for their antimicrobial properties. Many nitrothiophene compounds were previously reported to show high toxicity when considered for systemic administration. Therefore, highly active compounds sharing moderate toxicity could still be successfully applied as topical antimicrobial agents [23,24].

Our results show that systemic administration of KTU-286 could result in deleterious side effects associated with toxicity. The compound is mostly eliminated by kidneys and is metabolized via CYP3A4 cytochromes. In silico pharmacological modeling showed considerably low acute toxicity of KTU-286, and the major identified toxicity target was the mitochondrial toxicity pathway. The compound was predicted to show carcinogenicity, which is commonly described among a variety of thiophene derivatives [25]. Further experimental studies are needed to validate in silico predicted findings. Moreover, due to limited predicted absorption through the skin, KTU-286 could potentially be investigated as a topical antimicrobial agent for treatment and decolonization purposes.

Numbers of thiophene derivatives are known to have immunomodulatory activity. Therefore we employed in silico docking studies with various human proteins involved in enzymatic, regulatory, signaling, and response pathways. In silico docking studies revealed that KTU-286 interacts with human arachidonate 5-lipoxygenase (ALOX5), prostaglandin E synthase (PTGES), and interleukin-8 receptor B (CXCR2). Various compounds acing as inhibitors or activators of ALOX5, PTGES, and CXCR2 have been previously demonstrated as a strong immunomodulators exhibiting anti-inflammatory or immunomodulatory activity [26,27]. These identified targets interacting with KTU-286 are directly or indirectly involved in the leukotriene signaling pathway, suggesting possible immunomodulatory and anti-inflammatory properties of KTU-286.

The study of Masunari and Tavares reports the synthesis and biological activity of novel nifuroxazide-like compounds bearing nitrothiophene moieties with high antimicrobial activity against multidrug-resistant *S. aureus* [24]. The authors report that nitrofurane substitution with nitrothiophene results a series of compounds with high activity against MRSA, suggesting that the nitrothiophene moiety is an important pharmacophore for the anti-staphylococcal activity. Moreover, the study of Khambete et al. reports the synthesis of a nitrothiophene derivative showing low cytotoxicity and exceptional antimicrobial activity against *Mycobacterium* spp., suggesting the role of the nitrothiophene moiety as a leading pharmacophore important for the biological activity [23].

During our study, KTU-286 showed slight antimicrobial activity against *Mycobacterium abscessus* (MIC 64.0 µg/mL) but not *Mycobacterium bovis* BCG or *Mycobacterium smegmatis.* The previous studies of Rando et al. demonstrated the anti-mycobacterial activity of 5-nitro-2-thiophenecarbaldehyde *N*-((*E*)-(5-nitrothienyl)methylidene)hydrazone against *M. tuberculosis, M. kansasii*, and *M. avium* complex [22]. We have also shown that KTU-286 possesses a bactericidal activity that is highly selective and directed against *S. aureus*. Furthermore, this antimicrobial activity is not dependent on pre-existing antimicrobial resistance. Interestingly, KTU-286 showed better bactericidal activity than vancomycin against VRSA strains, making KTU-286 an attractive antimicrobial.

Biofilm production among *S. aureus* is important factor leading to the treatment failure among patients with chronic wound and prosthetic joint infections [26,28,29]. In comparison to planktonic forms, biofilm-associated *S. aureus* are virtually inaccessible to the host immune system and systemically administered antimicrobials. Even highly active agents such as vancomycin and daptomycin fail to ensure the full eradication of *S. aureus* biofilms from artificial surfaces, leading to relapsing infections [30,31]. Therefore, the potential drug–drug synergy of KTU-286 and other anti-staphylococcal agents should be explored to further increase the biofilm-targeting activity.

Thiophene derivatives were previously shown to have activity directed against biofilm integrity [32]. The study of Chorell et al. reports synthesis and characterization of thiophene-bearing compounds with antibiofilm activity. Incorporation of a thiophene moiety into the different positions of dihydrothiazolo-ring-fused 2-pyridones generated a series of compounds with activity against *Escherichia coli* biofilms. Therefore, various derivatives of thiophene could be potentially used as important compounds in the development of novel biofilm-targeting antimicrobial therapeutics [33]. We evaluated the activity of KTU-286 against biofilm integrity of *S. aureus* and explored whether the antibiofilm activity is dependent on the genetic resistance mechanisms. *S. aureus* biofilm disrupting activity of KTU-286 was observed at the concentration of four times the MIC. Anti-biofilm activity of KTU-286 could be further explored as a potential topical treatment option for the treatment of chronic wounds and implant-associated infections caused by highly resistant *S. aureus*. More studies are needed to better understand the antimicrobial activity of KTU-286 against other Gram-positive organisms and the drug–drug interactions of KTU-286 with other clinically available antimicrobials.

## 4. Materials and Methods

### 4.1. Bacterial Isolates and Culture Conditions

The antimicrobial activity of compound KTU-286 was evaluated by using a laboratory collection of bacterial and fungal pathogens (Table S1). The library of genetically defined isolates of *S. aureus* (*n* = 100) (obtained from the culture collection of the Institute of Infectious Diseases and Pathogenic Microbiology, Lithuania) was used for this study. Antimicrobial activity of KTU-286 against *S. aureus* was tested using *S. aureus* strains harboring SC*mecA* (*n* = 30) and *vanA* (*n* = 30) and pan-susceptible *S. aureus* isolates (*n* = 40).

Prior to the study, all strains were maintained in commercial cryopreservation systems at −80 °C. Bacterial strains were subcultured on Columbia Sheep Blood agar (Becton Dickenson, United States) or Middlebrook 7H9 media (7H9) (Becton Dickenson, Franklin Lakes, NJ, USA) for *Mycobacterium* spp. Fungal pathogens were cultured on Sabouraud Chloramphenicol agar (Liofilchem, Roseto degli Abruzz, Italy). Unless otherwise specified, all strains were further cultured in Cation-Adjusted Mueller–Hinton broth (CAMBH) for liquid cultures (Liofilchem, Italy) or Columbia Sheep Blood agar for solid cultures.

### 4.2. ADMET Prediction

The structure of KTU-286 was converted to SMILE structure and used for in silico ADMET. The absorption, distribution, metabolism, elimination, and toxicity (ADMET) parameters were predicted using pkCSM and ProTox-II software [34,35,36]. The pkCSM software provided information on following parameters: water solubility (log mol/L); Caco-2 intestinal permeability (cm/s); human intestinal absorption (%); human skin permeability (log Kp); activity as P-glycoprotein substrate; activity as P-glycoprotein I substrate and II inhibitor; volume of steady-state distribution (log L/kg); blood–brain barrier permeability (log BB); CNS permeability (log PS); metabolic interactions with cytochromes CYP2D6, CYP3A4, CYP1A2, CYP2C19, CYP2C9, CYP2D6, and CYP3A4; total renal clearance (log mL/min/kg); OCT2 substrate; AMES toxicity; maximal human tolerated dose (log mg/kg/day); human ERG I and II inhibition; rat oral acute toxicity (LD_50_); rat oral chronic toxicity (log mg/kg/day); hepatotoxicity; skin sensitization; *Tetrahymena pyriformis* toxicity (µg/L); and minnow toxicity (log mM) [35].

ProTox-II was used to predict immunotoxicity; cytotoxicity; and interactions with aryl hydrocarbon receptor (AhR), androgen receptor (AR), androgen receptor ligand binding domain (AR-LBD), aromatase, estrogen receptor alpha (ER), estrogen receptor ligand binding domain (ER-LBD), peroxisome proliferator activated receptor gamma (PPAR-Gamma), nuclear factor (erythroid-derived 2)-like 2/antioxidant responsive element (nrf2/ARE), heat shock factor response element (HSE), mitochondrial membrane potential (MMP), phosphoprotein p53, and ATPase family AAA domain-containing protein 5 (ATAD5) targets.

SwissTargetPrediction tool was employed to estimate the most probable macromolecular human targets of KTU-286.

All in silico ADMET predictions were made using default parameters.

### 4.3. Determination of Minimal Inhibitory Concentration (MIC)

The antimicrobial activity of KTU-286 was evaluated by using the broth microdilution method as suggested by Clinical Laboratory Standards Institute (CLSI), with brief modifications [37,38]. Briefly, KTU-286 was dissolved in dimethylsulfoxide (DMSO) to achieve a final concentration of 25 mg/mL. Series of dilutions were prepared in deep 96-well microplates to achieve 2× concentration of 0.25, 0.5, 1, 2, 4, 8, and 16 µg/mL using CAMHB (for bacteria), RPMI/MOPS (for fungal pathogens), or 7H9 (for *Mycobacterium* spp.). The microplates, containing series of dilutions, were inoculated with fresh cultures of each tested organism to achieve the final concentration of 5 × 10^4^ CFU of test organism in media containing 1% DMSO and 1× drug concentration in a volume of 200 µL per well. The inoculated wells, containing media with 1% DMSO, were used as a positive control. The microplates were sealed with plate-sealing tape and incubated at 35 ± 1 °C, for 18 ± 2 h, 24 h, and 48 h for fungal pathogens and 7 days for *Mycobacterium* spp.

After incubation, the plates were read using manual microplate viewer (Sensititre Manual Viewbox, United States). Minimal inhibitory concentration (MIC) was defined as lowest concentration (µg/mL) of a tested drug that fully inhibits growth of the test organism. All experiments were performed in duplicate with three technical replicates.

### 4.4. Concentration-Dependent S. aureus Growth Inhibition

Concentration-dependent *S. aureus* growth inhibition was evaluated by using the broth microplate dilution method, as described by CLSI, and a panel of *S. aureus* harboring genetically defined resistance mechanisms. Prior to experiments, 96-well flat-bottomed microplates, containing series of KTU-286 concentrations (prepared as 2× stock) dissolved in CAMBH containing 2% DMSO (as 2×), were prepared according to CLSI guidelines. Microplates were inoculated with a fresh culture of representative *S. aureus* strains carrying SC*mecA* (MRSA)*, vanA* (VRSA), and pan-susceptible (Pan-S) *S. aureus* to reach 5 × 10^4^ CFU of bacteria, 1× KTU-286 concentration (0.25, 0.5, 1, 2, 4, 8, 16 µg/mL), and 1% DMSO in a final volume of 200 µL. Inoculated microplates were incubated for 18 h at 35 ± 1 °C. After incubation, resazurin was added (20 µL of 22.2 mM stock) to each well to achieve final concentration of 2.2 mM, and microplates were further incubated for 2 h to quantify the surviving *S. aureus.*

After incubation, microplates were spun at 2000× *g* for 10 min; supernatant (100 µL) was removed and transferred to a new microplate, and OD_570nm_ was measured. Half-maximum activity (IC_50_) was calculated, as described elsewhere [39,40], using Prism GraphPad. All experiments were performed in triplicate.

### 4.5. Time-Kill Kinetics of KTU-286

The MIC concentration of KTU-286 (Table 7) was achieved in CAMBH containing final 1% DMSO concentration. The culture media with KTU-286 were inoculated with each representative strain of *S. aureus* (Pan-S, MRSA and VRSA) to reach approximately 1.5 × 10^4^ CFU/mL. Samples were incubated at 37 °C and 100 rpm for 12 h. The aliquots of 10 µL were taken at time points of 0, 1, 2, 3, 4, 5, 6, and 12 h. They were serially diluted in 96-well microplates and spotted on Columbia Sheep Blood agar. CAMBH containing 1% DMSO was used as a control. The colony-forming units were calculated. The experiment was performed in triplicate.

### 4.6. S. aureus Biofilm Formation Assay

The activity of KTU-286 **(2)** on the integrity of mature *S. aureus* biofilms was evaluated using the crystal violet biofilm assay as described by O’Toole with slight modifications [41]. Representative strains of *S. aureus* were cultured overnight in a rotary shaker at 37 °C and 200 rpm using 5 mL of CAMBH. The overnight cultures of test strains were adjusted to reach approximately OD_600 nm_ = 0.4 (corresponding to 4.0 × 10^8^ CFU/mL). To initiate the growth of *S. aureus* biofilms, 96-well flat-bottomed microplates were inoculated with 4.0 × 10^7^ CFU/well of each *S. aureus* strain in the final volume of 100 µL. *S. aureus* biofilms were cultured for 48 h at 37 °C. After cultivation, media containing planktonic *S. aureus* were aspirated, and then fresh CAMBH (100 µL) was added to each well. KTU-286 was added as 2× concentrate to achieve 0.5 MIC, MIC, 2× MIC and 4× MIC concentrations of KTU-287 in CAMBH with 1% DMSO in individual wells. Wells containing only 1% DMSO were used as negative control. Biofilms with test compound were further incubated for 24 h at 37 °C.

After exposure to the test compound, media were aspirated; biofilms were washed with sterile phosphate-buffered saline (PBS) and fixed using 4% paraformaldehyde overnight at room temperature. Paraformaldehyde was removed, and biofilms were stained using 0.5% crystal violet at room temperature for 30 min. Stained microplates were washed five times using deionized water and dried overnight at 37 °C, and absorbed crystal violet was solubilized using 30% acetic acid. Biofilm production was quantified by measuring absorption at OD_550_. Experiments were performed in triplicate.

### 4.7. Statistical Analysis

The results are expressed as mean ± standard deviation (SD). Statistical analyses were performed with Prism (GraphPad Software, San Diego, CA, USA), using Kruskal–Wallis test and two-way ANOVA. *p* < 0.05 was accepted as significant.

## 5. Conclusions

KTU-286 was demonstrated to have in vitro antimicrobial activity against multidrug-resistant *S. aureus* with defined mechanisms of resistance. Exceptional activity against *S. aureus* biofilms makes KTU-286 an attractive antimicrobial, simultaneously targeting virulence and growth, with promising topical application properties. Further studies are needed for better understanding of the safety, synergistic relationships, and therapeutic potency of KTU-286.

## Figures and Tables

**Table 1:** The in silico pharmacological permeability characteristics of KTU-286 predicted using pkCSM software.

Pharmacological Test	KTU-286	Vancomycin
Water solubility (log mol/L)	−4.724	−2.892
BBB permeability (log BB)	−1.09	−3.486
Caco2 permeability (cm/s)	0.932	−1.963
CNS permeability (log PS)	−2.249	−6.619
Human intestinal absorption (% of absorption)	94.139	0
Human skin permeability (log Kp)	−2.642	−2.735
P-glycoprotein I inhibitor	No	No
P-glycoprotein II inhibitor	No	No
P-glycoprotein substrate	No	Yes

BBB, blood–brain barrier; CNS, central nervous system.

**Table 2:** The in silico pharmacological excretion characteristics of KTU-286 predicted using pkCSM software.

Pharmacological Test	KTU-286	Vancomycin
VDss (log L/Kg)	−0.011	−0.287
Total renal clearance (log mL/min/Kg)	0.479	−1.043
Renal OCT2 substrate	No	No

VD_ss_, steady-state volume distribution; OCT2, organic cation transporter 2. VD_ss_ is defined as the equivalent volume into which a given dose of drug is apparently distributed within the body; as such, it includes the extent to which the drug is bound to tissue and/or plasma proteins.

**Table 3:** The in silico pharmacological prediction of KTU-286 interaction with major human-cytochrome-mediated metabolism systems was estimated using pkCSM software.

Interaction with Cytochrome	KTU-286	Vancomycin
CYP2D6 substrate	No	No
CYP3A4 substrate	Yes	No
CYP1A2 inhibition	Yes	No
CYP2C19 inhibition	Yes	No
CYP2C9 inhibition	Yes	No
CYP2D6 inhibition	No	No
CYP3A4 inhibition	No	No

**Table 4:** The toxicological parameters of KTU-286 were predicted using ProTox and pkCSM software.

Toxicological Test	KTU-286	Vancomycin
AMES toxicity	Yes	No
Hepatotoxicity	No	No
Human ERG I	No	No
Human ERG II	No	Yes
Immunotoxicity	No	No
Maximal human tolerated dose (log mg/kg/day)	0.164	0.439
Minnow toxicity (log mM)	0.222	13.849
Murine LD50 (mg/kg)	750	1500
Rat oral acute toxicity (LD50) (mol/kg)	2.748	2.482
Rat oral chronic toxicity (log mg/kg/day)	1.149	9.152
Skin sensitization	No	No
*T. pyriformis* toxicity (µg/L)	2.125	13.849

**Table 5:** The prediction of KTU-286 in silico interaction with major human toxicity targets.

Toxicity Pathway	Toxicity Target	Prediction Result	Probability *
Nuclear receptor signaling pathways	Aryl hydrocarbon receptor (AhR)	Inactive	0.53
Nuclear receptor signaling pathways	Androgen receptor (AR)	Inactive	0.97
Nuclear receptor signaling pathways	Androgen receptor ligand-binding domain (AR-LBD)	Inactive	0.95
Nuclear receptor signaling pathways	Aromatase	Inactive	0.92
Nuclear receptor signaling pathways	Estrogen receptor alpha (ER)	Inactive	0.67
Nuclear receptor signaling pathways	Estrogen receptor ligand-binding domain (ER-LBD)	Inactive	0.81
Nuclear receptor signaling pathways	Peroxisome proliferator-activated receptor gamma (PPAR-Gamma)	Inactive	0.88
Stress response pathways	Nuclear factor (erythroid-derived 2)-like 2/antioxidant responsive element (nrf2/ARE)	Inactive	0.74
Stress response pathways	Heat shock factor response element (HSE)	Inactive	0.74
Stress response pathways	Mitochondrial membrane potential (MMP)	Active	0.67
Stress response pathways	Phosphoprotein (tumor suppressor) p53	Inactive	0.78
Stress response pathways	ATPase family AAA domain-containing protein 5 (ATAD5)	Inactive	0.78

* The probability on the left-hand side gives a confidence estimate for the prediction.

**Table 6:** The distribution of minimal inhibitory concentration (MIC) values of KTU-286 among *Staphylococcus aureus* (*n* = 100) strains with defined resistance determinants.

Resistance Mechanisms	Major Resistance Determinant	No. of Tested Isolates	No. of *S. aureus* Strains (%) per KTU-286 Concentration (µg/mL)							MIC_50_^1^	MIC_90_^2^	VAN MIC
			0.25	0.5	1.0	2.0	4.0	8.0	16.0			
*S. aureus* Pan-S	*blaZ*	40	0 (0.0)	15 (37.5)	18 (45.0)	7 (17.5)	0 (0.0)	0 (0.0)	0 (0.0)	0.5	1.0	0.5–1.0
*S. aureus* MRSA	SC*mecA*	30	0 (0.0)	0 (0.0)	6 (20.0)	2 (6.7)	12 (40.0)	4 (13.3)	6 (20.0)	1.0–2.0	4.0	1.0–2.0
*S. aureus* VRSA	*vanA*	30	0 (0.0)	0 (0.0)	0 (0.0)	0 (0.0)	30 (100.0)	0 (0.0)	0 (0.0)	4.0	4.0	8.0–320

^1.^ MIC_50_ was defined as a minimum concentration (µg/mL) of KTU-286 inhibiting growth of 50% tested *S. aureus* isolates; ^2.^ MIC_90_ was defined as a minimum concentration (µg/mL) of KTU-286 inhibiting growth of 90% tested *S. aureus* isolates; VAN MIC, minimal inhibitory concentration (MIC) of vancomycin.

**Table 7:** Minimal inhibitory concentration (MIC) and IC_50_ of KTU-286 and vancomycin among selected representative strains of *Staphylococcus aureus* harboring genetically defined resistance mechanisms.

Organism	Strain No.	Phenotype	Resistance Mechanisms	KTU-286 MIC	IC_50_	VAN MIC
*S. aureus*	SA-1001	Pan-S	*blaZ*	2.0	0.46	0.5
*S. aureus*	ME-311	MRSA	SC*mecA, ermA*	8.0	2.802	1.0
*S. aureus*	VA13	VRSA	*vanA, aac(6′)-aph(2″), tetK*	4.0	1.697	32.0

VAN MIC, minimal inhibitory concentration of vancomycin (µg/mL).

## Supplementary Materials

The following are available online at https://www.mdpi.com/2079-6382/9/9/612/s1, title, Table S1: The bacterial and fungal isolates used for the in vitro activity testing. Table S2: The in silico predictions of human proteins interacting with KTU-286. The in silico predictions were computed using SwissTargetPrediction tool.
