# Supplementary material for: Synthesis, ADMET Properties, and In Vitro Antimicrobial and Antibiofilm Activity of 5-Nitro-2-thiophenecarbaldehyde N-((E)-(5-Nitrothienyl)methylidene)hydrazone (KTU-286) against Staphylococcus aureus with Defined Resistance Mechanisms
Source: Antibiotics (Basel). 2020 Sep 17;9(9):612. doi: 10.3390/antibiotics9090612 (PMC7558474; doi:10.3390/antibiotics9090612)
Supplement: Supplementary file 1 [file antibiotics-09-00612-s001.pdf]

Table S1. The bacterial and fungal isolates used for the in vitro activity testing.

Microorganism	Strain No	Feature	KTU-286 MIC (µg/mL)
Acinetobacter baummanii	ATCC 17978	Reference strain	128 <
Aspergillus flavus	12B	Clinical isolate	128 <
Aspergillus fumigatus	14	Clinical isolate	128 <
Candida albicans	ATCC 10231	Type strain	128 <
Candida auris	CAU-1	Clinical isolate	128 <
Cuninghamella corymbifera	CC1	Clinical isolate	128 <
Escherichia coli	10025	Mcr-1	128
Escherichia coli	DH5a	Pan-S	128
Klebsiella pneumoniae	3122	bla_{KPC}	128 <
Klebsiella pneumoniae	4141	bla_{NDM-1}	128 <
Mycobacterium abscesus	IP-K01	Clinical isolate	64
Mycobacterium bovis	BCG	Type strain	128 <
Mycobacterium smegmatis	mc^{2}155	Type strain	128 <
Pseudomonas aeruginosa	3691	AmpC	128 <
Pseudomonas aeruginosa	3656	Pan-S	128 <
Pseudomonas aeruginosa	3647	Efflux pump overexpression	128 <
Pseudomonas aeruginosa	3619	OPRD	128 <
Staphylococcus aureus	875	MRSA	1

Table S2. The In silico predictions of human proteins interacting with KTU-286. The in silico predictions were computed using SwissTargetPrediction tool.

Identified target	Common name	Uniprot ID	ChEMBL ID	Target Class	Probability
Arachidonate 5-lipoxygenase	ALOX5	P09917	CHEMBL215	Oxidoreductase	0.100578902
Prostaglandin E synthase	PTGES	O14684	CHEMBL5658	Enzyme	0.100578902
Interleukin-8 receptor B	CXCR2	P25025	CHEMBL2434	Family A G protein-coupled receptor	0.100578902
Cyclooxygenase-2	PTGS2	P35354	CHEMBL230	Oxidoreductase	0.100578902
Carboxylesterase 2	CES2	O00748	CHEMBL3180	Enzyme	0.100578902
Poly [ADP-ribose] polymerase 2	PARP2	Q9UGN5	CHEMBL5366	Enzyme	0.100578902
Tankyrase-2	TNKS2	Q9H2K2	CHEMBL6154	Enzyme	0.100578902
Tankyrase-1	TNKS	O95271	CHEMBL6164	Enzyme	0.100578902
Trace amine-associated receptor 1 (by homology)	TAAR1	Q96RJ0	CHEMBL5857	Family A G protein-coupled receptor	0.100578902
Death-associated protein kinase 3	DAPK3	O43293	CHEMBL2468	Kinase	0.100578902
Carbonic anhydrase II	CA2	P00918	CHEMBL205	Lyase	0.100578902
Carbonic anhydrase I	CA1	P00915	CHEMBL261	Lyase	0.100578902
Carbonic anhydrase IX	CA9	Q16790	CHEMBL3594	Lyase	0.100578902

Epidermal growth factor receptor erbB1	EGFR	P00533	CHEMBL203	Kinase	0.100578902
Acyl coenzyme A:cholesterol acyltransferase	CES1	P23141	CHEMBL2265	Enzyme	0.100578902
Phosphomannomutase 2	PMM2	O15305	CHEMBL1741162	Enzyme	0.100578902
Cyclin-dependent kinase 5/CDK5 activator 1	CDK5R1 CDK5	Q15078 Q00535	CHEMBL1907600	Kinase	0.100578902
Cyclin-dependent kinase 2/cyclin E1	CCNE1 CDK2	P24864 P24941	CHEMBL1907605	Kinase	0.100578902
Mannose-6-phosphate isomerase	MPI	P34949	CHEMBL2758	Isomerase	0.100578902
Glutathione S-transferase Pi	GSTP1	P09211	CHEMBL3902	Enzyme	0.100578902
Phospholipase C-gamma-2	PLCG2	P16885	CHEMBL4100	Hydrolase	0.100578902
Glutathione S-transferase Mu 2	GSTM2	P28161	CHEMBL4589	Enzyme	0.100578902
Phosphoethanolamine/phosphocholine phosphatase	PHOSPHO1	Q8TCT1	CHEMBL6113	Enzyme	0.100578902
Leukocyte elastase	ELANE	P08246	CHEMBL248	Protease	0.100578902
Cathepsin D	CTSD	P07339	CHEMBL2581	Protease	0.100578902
Adenosine A3 receptor	ADORA3	P0DMS8	CHEMBL256	Family A G protein-coupled receptor	0.100578902
Thrombin	F2	P00734	CHEMBL204	Protease	0.100578902
Complement C1r	C1R	P00736	CHEMBL4611	Protease	0.100578902
Beta-chymotrypsin	CTRB1	P17538	CHEMBL4796	Protease	0.100578902
Glycogen synthase kinase-3 beta	GSK3B	P49841	CHEMBL262	Kinase	0.100578902
Toll-like receptor (TLR7/TLR9)	TLR9	Q9NR96	CHEMBL5804	Toll-like and Il-1 receptors	0.100578902
